# Synthesis of zinc oxide nanorods or nanotubes on one side of a microcantilever

**DOI:** 10.1098/rsos.180510

**Published:** 2018-08-08

**Authors:** Laurent Schlur, Jeremy Ramos Calado, Denis Spitzer

**Affiliations:** Nanomatériaux pour les Systèmes Sous Sollicitations Extrêmes (NS3E), UMR 3208 ISL/CNRS/UNISTRA, French-German Research Institute of Saint-Louis, 5, rue du Général Cassagnou, 68300 Saint-Louis, France

**Keywords:** nanostructured microcantilevers, ZnO nanorods, ZnO nanotubes, synthesis improvement, sensor

## Abstract

Cantilevers are really promising sensitive sensors despite their small surface. In order to increase this surface and consequently their sensitivity, we nanostructured them with zinc oxide (ZnO) nanorods or nanotubes having a diameter of approximately 100 nm and a length of 1 µm. The nanostructure growth was first optimized on a silicon wafer and then transferred to the cantilevers. The ZnO nanorods were grown in an autoclave. The centre of the nanorods was dissolved in order to obtain nanotubes. The dissolution conditions were optimized in order to have the longest etching depth. After 1.25 h in a dissolution solution containing 0.75 wt% of NH_3(aq)_ and 0.75 wt% of cetyltrimethyl ammonium bromide, the longest etching depth was obtained. After the transfer of the syntheses to the cantilevers, nanorods/nanotubes grew on both sides of the cantilever, which prevents the reflection of the laser allowing the resonance frequency measurement. A masking procedure was developed in order to avoid the growth on one face of the cantilever of zinc oxide nanostructures. As far as the authors are concerned, for the first time, zinc oxide nanotubes were synthesized on only one face of cantilevers with optical readout.

## 1. Introduction

One-dimensional (1D) inorganic nanostructures, such as nanorods and nanotubes, have been intensively studied over the last years due to their significance in basic scientific research and potential technological applications [1–9]. Zinc oxide has been one of the most studied 1D nanostructured materials because of its interesting physical and chemical properties. Zinc oxide has a direct wide band gap of 3.37 eV, a large exciton binding energy of 60 meV at room temperature and piezoresistive properties [10,11]. 1D ZnO structures have several applications such as sensors [12–14], solar cells [15,16], photocatalysts [17] and field emission devices [18].

The growth of ZnO nanorods arrays on substrates can be obtained either using vapour phase syntheses or in solutions [11]. The former requires expensive equipment, high temperatures and time-consuming procedures [19,20] whereas the latter is easy to perform at low temperatures [21]. The most often used ZnO nanorod solution syntheses are the hydrothermal growth [22,23], the electrochemical way [24] or by wet chemical synthesis [25].

Zinc oxide nanotube arrays are almost always obtained by the dissolution of the centre of the nanorods previously synthesized. This dissolution can be done electrochemically [24], but the chemical dissolution is mostly used. The chemical dissolution can be done in an acidic (HCl) or a basic (KOH) solution as zinc oxide is amphoteric [26–28]. Two hypotheses can explain the selective dissolution of the centre of the nanorods with no attack of the lateral faces. Firstly, the (0001) zinc oxide metastable plans have a higher surface energy than the lateral plans which are consequently more stable [29,30]. Secondly, the (0001) plans present more defects, which promotes their dissolution [30]. Wang *et al.* added a surfactant (cetyl trimethylammonium bromide) to the dissolution solution [31]. This molecule is well known to be fixed to the (1000) plans and consequently protect the wall of the 1D structure during the dissolution of the nanorods into nanotubes [32–35]. The etching agent used is ammonia solution.

In this article, we adapt the etching process developed by Wang *et al.* for nanorods with a diameter around 100 nm. Indeed, Wang *et al*. used only rods with a diameter higher than 200 nm [31]. This diameter difference obliged the authors to change the synthesis conditions totally, so this article is not just an adaptation of Wang's synthesis. We were obliged to modify the concentrations and the reaction times in order to increase the etching depth of nanotubes. The optimized nanotubes are then synthesized on the surface of commercial atomic force microscopy (AFM) cantilevers, which can be used for the detection of amine molecules [14]. The presence of nanostructures on the surface of the cantilever should increase the sensitivity of the sensor as the cantilever surface is improved [2]. As the surface developed by the nanotubes is higher than that of the nanorods (for one given length and diameter), the sensitivity of the sensor covered by nanotubes should be higher than the same sensor covered by nanorods. The selectivity and the sensitivity of the sensor can also be improved, if the material of the nanostructures has naturally a good affinity with the molecules which have to be detected [2,36]. Killinc *et al.* already synthesized ZnO nanotubes on home-made nickel cantilevers [14]. Our objective is to obtain nanotubes which are more open than Killinc's nanotubes and to use a synthesis which can be adapted to every silicon commercial cantilever, which was not the case before this publication.

## 2. Experimental section

### 2.1. Zno thin layer

**Figure 1.**
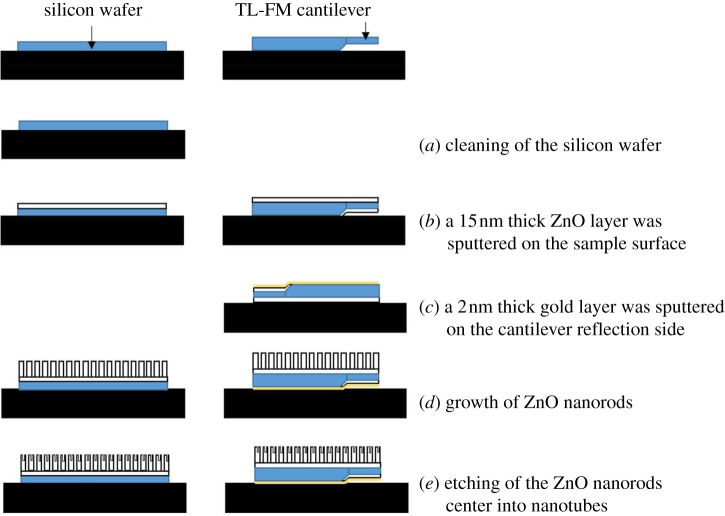
Fabrication process of the ZnO nanostructured wafers and cantilevers.

The fabrication processes of nanostructured wafers and cantilevers are shown in figure 1.

Prior to the sputtering process, the silicon wafer (1 × 1 cm², one side polished), purchased from Siegert Consulting e.K, was successively cleaned in acetone, ethanol and water under sonication and subsequently dried under N_2_ atmosphere (figure 1*a*). The substrate was then submitted for 15 min at 50°C to an UV-Ozone treatment (Novascan PSD-UVT device). Afterwards, the cleaned wafer was put in a magnetron sputtering system (HHV Auto 360) equipped with a zinc oxide target (99.999%). The chamber was pumped down to 1 × 10^−5^ mbar. Deposition was realized at a pressure of 2 × 10^−3^ mbar and the magnetron power density was 0.66 W cm^−2^. The sputtered zinc oxide layer thickness was 15 nm (figure 1*b*).

The zinc oxide sputtering process was not modified when the wafer was replaced by a TL-FM tipless cantilever, except for the fact that the cantilever was not cleaned before use. These cantilevers, purchased from NanoAndMore, have a length of 225 ± 10 µm, a width of 28 ± 7.5 µm, a thickness of 3.0 ± 1 µm and a resonant frequency between 45 and 115 kHz.

### 2.2. Zno nanorods

Ethylenediamine (C_2_H_4_(NH_2_)_2_, purity ≥ 99%) and zinc acetate dihydrate (Zn(CH_3_COO)_2_·2H_2_O, purity ≥ 98%), each purchased from Sigma Aldrich, were the two reactants used in the ZnO nanorod growth.

ZnO nanorods were grown in an autoclave (figure 1*d*). In a typical procedure, 10 ml of freshly prepared solution of ethylenediamine (20 vol%) was mixed with 24 ml of an aqueous zinc acetate dihydrate solution (0.72 mol l^−1^). This solution was then poured into a home-made Teflon lined autoclave (*V* = 80 ml). The wafer covered by 15 nm of ZnO was suspended horizontally upside-down in the autoclave. The latter was heated at 65°C for 2 h in order to avoid the deposition of ZnO particles on the surface of the nanorods. After the synthesis, the wafer is washed with distilled water and dried with a N_2_ flow.

### 2.3. Zno nanotubes

Ammonia solution (NH_3(aq)_, 30%) and cetyltrimethyl ammonium bromide (CTAB, purity ≥ 99%), purchased from Roth and Sigma Aldrich, respectively, were used to dissolve the centre of the ZnO nanorods into nanotubes.

Zinc oxide nanotubes were prepared by submitting the previously synthesized nanorods to a wet chemical treatment based on the method developed by Wang *et al.* [31] (figure 1*e*). In a typical procedure, an aqueous solution of 90 ml containing 0.5 wt% of ammonia (NH_3(aq)_) and 0.5 wt% of CTAB was poured into a beaker. The wafer covered with ZnO nanorods was placed horizontally upside-down in the beaker for 3.5 h at room temperature.

### 2.4. Characterization

The morphology and the size of the nanostructures were studied by scanning electron microscopy (SEM), using a FEI Nova NanoSEM 450 equipped with a field emission gun. X-ray diffraction (XRD) measurements were performed on a Bruker D8 Advance diffraction spectrometer (Cu K*α*_1_ = 0.15406 nm) with a voltage of 40 kV and an intensity of 40 mA. The zinc oxide layer surface morphology and roughness were determined by AFM using the tapping mode of a Veeco Nanoscope IV Multimode AFM. TEM images were recorded on a JEOL, ARM200CF (Tokyo, Japan), with a nominal point resolution of 0.8 A at Scherzer defocus.

## 3. Results and discussion

### 3.1. Zno thin layer and ZnO nanorods

**Figure 2.**
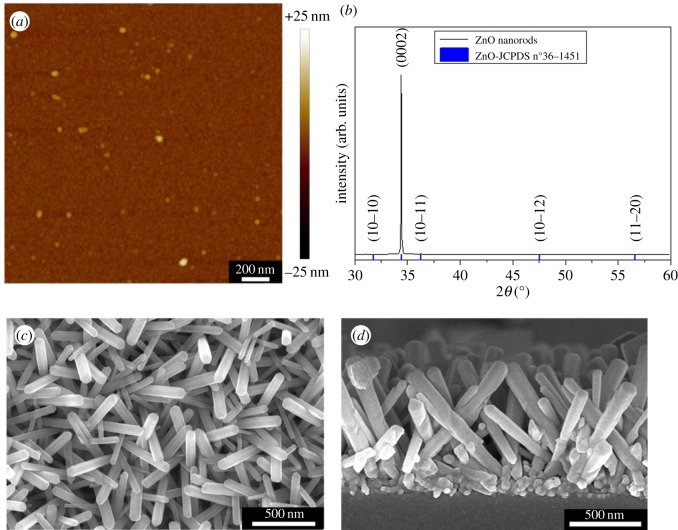
(*a*) AFM image of the 15 nm thick ZnO seed layer sputtered on the wafer surface. (*b*) X-ray diffraction pattern recorded on the ZnO nanorods grown on a silicon wafer covered by thin zinc oxide layer. SEM (*c*) top and (*d*) side view of the same nanorods.

The quality of the seed layer is critical to obtain homogeneous and oriented nanorods. The repartition of the zinc oxide seed layer is homogeneous and on the wafer surface despite the really low thickness of the layer (figure 2*a*). The seeds have a diameter of 23.6 ± 4.6 nm. A RMS roughness of 1.15 ± 0.12 nm has also been estimated thanks to AFM measurements.

The wafer covered by a 15 nm thick zinc oxide layer is put in the autoclave in order to synthesize zinc oxide nanorod arrays. Figure 2*c*,*d* shows that nanorods are present on the wafer surface. These nanorods are oriented upwards. Their size is relatively homogeneous. The nanorod diameter is equal to 114.17 ± 20.77 nm. In the following text, we will continue to call them nanorods even if the diameter is slightly higher than 100 nm. The taller ones have a homogeneous length of 1.05 ± 0.12 µm. No impurities are visible on the surface of the nanorods which confirms the results obtained by Schlur *et al.* [15]. The analysis of the X-ray diffraction pattern proves the presence of zinc oxide at the wafer surface (figure 2*b*). Zinc oxide crystallizes in the wurzite structure. The high intensity of the (0002) reflection confirms that the ZnO nanorods are orientated perpendicularly to the substrate.

### 3.2. Zno nanotubes

**Figure 3.**
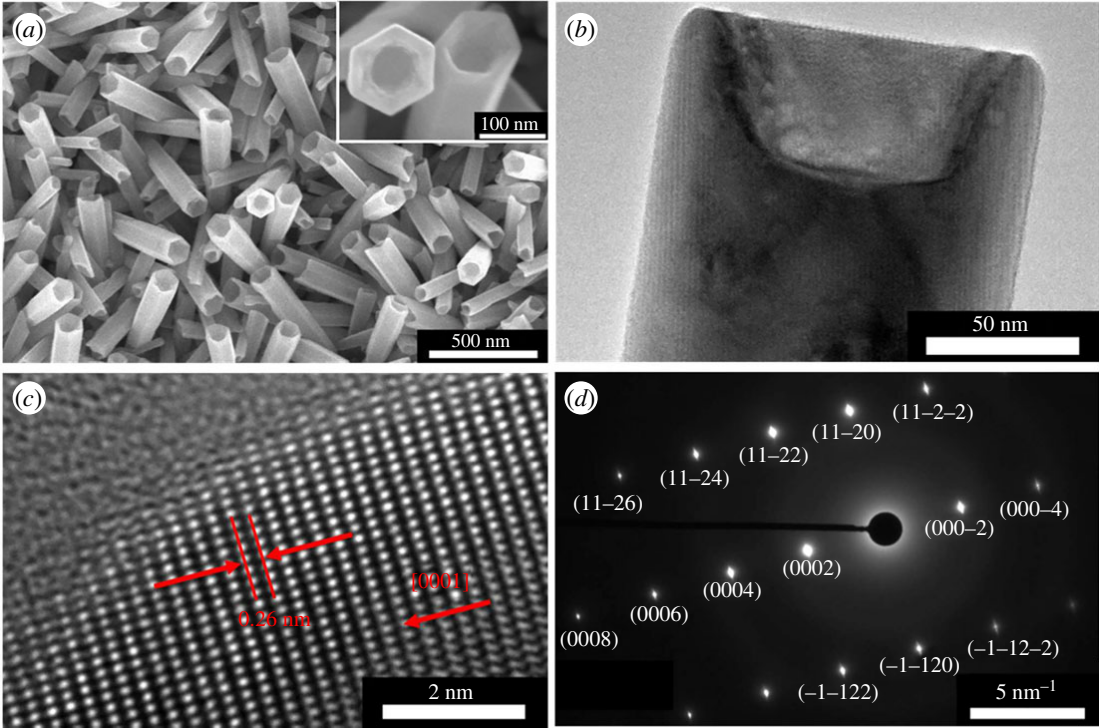
(*a*) SEM top view of nanotube arrays obtained by the etching of nanorods in a solution containing 0.5 wt% NH_3(aq)_ and 0.5 wt% CTAB (time: 3.5 h). Inset: zoom of the SEM top view. (*b*) Low resolution and (*c*) high resolution TEM picture of a single nanotube obtained in the same conditions as previously. (*d*) Electronic diffraction associated with picture (*c*).

The nanorod etching process used to obtain nanotubes is identical to the method developed by Wang *et al.* [31]. Figure 3 shows the obtained results.

The nanotubes are homogeneously etched and the walls seem not to be damaged. The nanotubes have an external diameter of 124.86 ± 16.81 nm which is close to the value obtained for the nanorods. Figure 3*b* shows that the nanotubes are not fully etched. The etching depth is 50.27 ± 0.54 nm which corresponds only to 4.8% of the length of the nanorods having a mean length of 1.05 ± 0.12 µm. Even if the nanostructures are not fully hollow, these types of nanostructures are called ‘nanotubes' in all the articles. The (0001) plans are clearly visible on the high-resolution TEM picture and no defects seem to be present. The electronic diffraction confirms the monocrystallinity of the nanotubes.

The CTAB surfactant is removed by calcination. Electronic supplementary material, figure S1 shows that all CTAB is evaporated for temperatures higher than 500°C. So to remove all the CTAB surfactants, the samples are annealed to 600°C.

**Figure 4.**
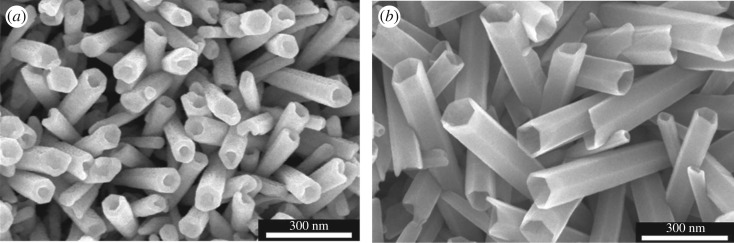
SEM top view of the nanotubes obtained in an etching solution containing: (*a*) 0.75 wt% of NH_3(aq)_ and 1.5 wt% of CTAB; (*b*) 0.75 wt% NH_3(aq)_ and 0.25 wt% of CTAB.

The nanorods are not fully etched contrary to the results obtained by Wang *et al.* [31]. In order to understand why and also to increase the etching depth of the nanorods, the NH_3(aq)_ ([NH_3(aq)_]) and CTAB ([CTAB]) concentrations have been modified independently. The [NH_3(aq)_] value was varied between 0.5 and 2 wt% and the [CTAB] between 0.25 and 1.5 wt%. The more representative results are visible in figure 4.

Figure 4*a* shows that some nanorods are not etched. A [CTAB] concentration that is too high compared to the [NH_3(aq)_] prevents a deep chemical etching. Indeed, an excess of surfactant obstructs the (0001) plans and consequently stopped the etching. An excess of NH_3(aq)_ (or a too low concentration of CTAB) is at the origin of an incomplete nanotube wall protection. Indeed, some walls are damaged during the etching (figure 4*b*). These results prove that an important difference between the NH_3(aq)_ concentration and the CTAB concentration does not allow the proper etching of nanorods into nanotubes, that is why in the following part the [NH_3(aq)_]/[CTAB] is kept equal to 1.

**Figure 5.**
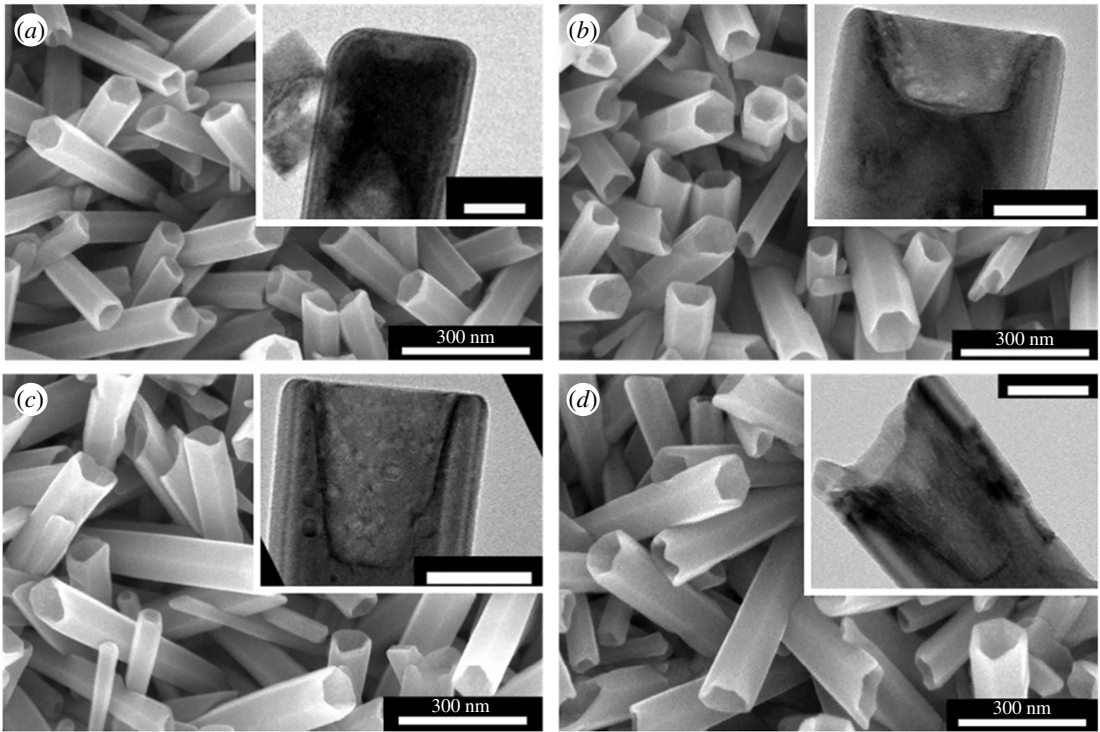
SEM top view of nanotubes obtained after an etching time of (*a*) 1.75, (*b*) 3.5, (*c*) 5.25 and (*d*) 7 h. The etching solution contains 0.5 wt% of NH_3_ and 0.5 wt% of CTAB. Inset: TEM images of the nanotubes (scale bar: 50 nm).

The influence of the reaction time on the etching depth was tested. The CTAB and NH_3(aq)_ concentrations were both 0.5 wt%. The different tested reaction times were 1.75, 3.5, 5.25 and 7 h. Figure 5 presents the obtained results.

**Figure 6.**
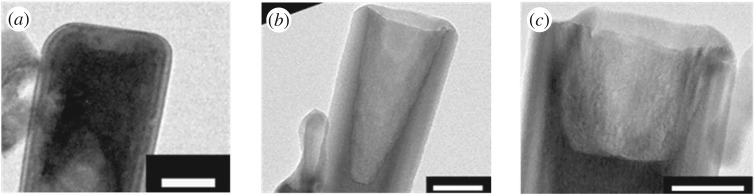
TEM images of isolated nanotubes obtained after 1.75 h of etching in solutions containing (*a*) 0.5 wt% of NH_3(aq)_ and CTAB, (*b*) 0.75 wt% of NH_3(aq)_ and CTAB, (*c*) 1 wt% of NH_3(aq)_ and CTAB. Scale bar 50 nm.

All the obtained nanotubes are etched uniformly whatever the dissolution time. An estimation of the etching depth is possible with the TEM images (inset, figure 5). The etching depth increases with the reaction time. The values are listed in table 1. For a reaction time of 7 h, only 15.7% of the total length of the tube is etched. We have tried to continue to increase the etching time in order to etch all the length of the tubes, but for longer reaction times the nanotube array peels off the wafer. So, the adhesion of the nanotubes on the wafer surface does not allow an increase in the etching reaction time. In order to continue increasing the etching depth of the tubes, the concentrations of the etching agent and of the surfactant were modified proportionally, i.e. the [NH_3(aq)_] was modified, while the [NH_3(aq)_]/[CTAB] kept unchanged and equal to 1. The results are visible in figure 6.

**Table 1.** Evolution of the nanotubes length, diameter and etching depth with the CTAB concentration, with the NH_3(aq)_ concentration and with the etching time. All the nanotubes were grown on silicon wafers.

[CTAB] (wt%)	[NH_3(aq)_] (wt%)	etching time (h)	nanotube length (µm)	nanotube diameter (nm)	etching depth (nm)	etching depth (%)
0.5	0.5	1.75	1.01 ± 0.10	121.23 ± 1.51	22.15 ± 0.87	2.2
0.5	0.5	3.5	1.05 ± 0.12	123.99 ± 0.54	50.27 ± 0.54	4.8
0.5	0.5	5.25	1.02 ± 0.10	117.76 ± 0.59	103.15 ± 0.89	10.1
0.5	0.5	7	0.94 ± 0.7	128.05 ± 1.01	147.67 ± 3.40	15.7
0.75	0.75	1.75	1.05 ± 0.13	108.87 ± 0.66	165.97 ± 0.41	15.9
1.0	1.0	1.75	1.02 ± 0.15	128.05 ± 1.01	100.39 ± 1.66	9.6
0.75	0.75	3.5	0.93 ± 0.16	116.58 ± 22.56	120.79 ± 3.4	13.0
0.75	0.75	5.25	0.76 ± 0.10	119.26 ± 22.99	108.55 ± 44.04	14.3

The chemical etching is more efficient for CTAB and NH_3(aq)_ concentrations of 0.75 wt% than for concentrations of 0.5 and 1 wt% (table 1). Indeed, for a reaction time of 1.75 h, the etching depth is equal to 165.97 ± 0.41 nm (15.88%), which is deeper than the results obtained for an etching of 7 h with a concentration of 0.5 wt% of NH_3(aq)_ and 0.5 wt% of CTAB. For concentrations higher or equal to 1 wt% of NH_3(aq)_ and CTAB, the etching depth value does not increase: 100.39 ± 1.66 nm (9.8%). This evolution can be explained by a high CTAB concentration. The CTAB molecules are fixed on the (0001) plans, which prevents a deep etching of the nanotubes.

**Figure 7.**
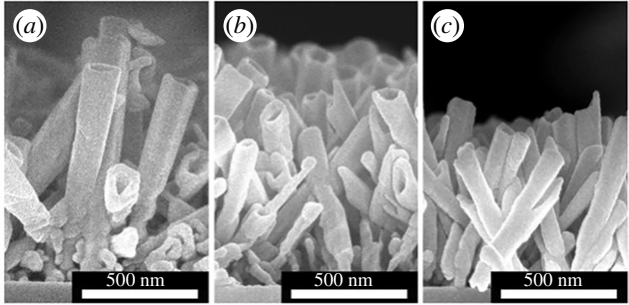
SEM side view of the ZnO nanotubes obtained after the dissolution of the nanorods during (*a*) 1.75 h, (*b*) 3.5 h and (*c*) 5.25 h in a solution containing 0.75 wt% of CTAB and 0.75 wt % of NH_3(aq)_.

In order to continue increasing etching depths, the etching time was increased for NH_3(aq)_ and CTAB concentrations equal to 0.75 wt%. For reaction times of 3.5 and 5.25 h, the etching depths are 120.79 ± 3.4 nm and 108.55 ± 44.04 nm, respectively (table 1). So far, NH_3(aq)_ and CTAB concentrations are equal to 0.75 wt%, the etching depth decreases when the reaction time increases, contrary to the results obtained for [NH_3(aq)_] = [CTAB] = 0.5 wt%. To explain this phenomenon, the evolution of the nanotube length was studied (figure 7).

The nanotube length decreases with the reaction time in solutions containing 0.75 wt% of NH_3(aq)_ and 0.75 wt% of CTAB. For such concentrations, the etching affects not only the (0001) plans but also the nanotube walls. The dissolution of the walls explains the decrease in the etching depth during the time.

After having changed several parameters, the best nanotube etching depth has been obtained for 1.75 h of dissolution in an aqueous solution containing 0.75 wt% of NH_3(aq)_ and 0.75 wt% of CTAB. In these conditions, the etching depth represents 15.9% of the nanostructure length. These results are far from the fully etched nanotubes obtained by Wang *et al.* [31]. In order to understand why, we changed the nanorod synthesis and did exactly the same protocol than Wang *et al.* [31]. The nanorods are then etched for 3.5 h in 90 ml of an aqueous solution containing CTAB (0.5 wt%) and NH_3(aq)_ (0.5 wt%). These conditions were used by Wang *et al.* [31]. Electronic electronic supplementary material, figure S3 shows the obtained result. The nanotubes are not fully etched. So, etching problems encountered in this paper are not due to the fact that the ZnO nanorods were synthesized with zinc acetate dihydrate and ethylenediamine instead of zinc dichloride and ammonia solution.

The partial etching of the nanorods obtained with ZnCl_2_ and NH_(aq)_ is surprising as Wang *et al.* obtained fully etched nanostructures [31]. This difference can be due to the fact that in this publication the diameter of the nanorods (156.6 ± 17.8 nm) is thinner than in the publication used as reference (≈200 nm), which complicates the dissolution of the centre of nanorods into nanotubes. The purity of the reactants can also explain the difference observed. The defects in the crystal structure of zinc oxide which can be due to the presence of impurities play an important role in the dissolution of the centre of the nanorods. Indeed, if the top of the nanorods has not enough defects, the dissolution is not possible [30]. One of these two hypotheses or both can also explain why it is not possible to obtain fully etched nanotubes, when the nanorods are synthesized with zinc acetate dihydrate and ethylenediamine.

### 3.3. Cantilevers nanostructured with ZnO nanorods/nanotubes

**Figure 8.**
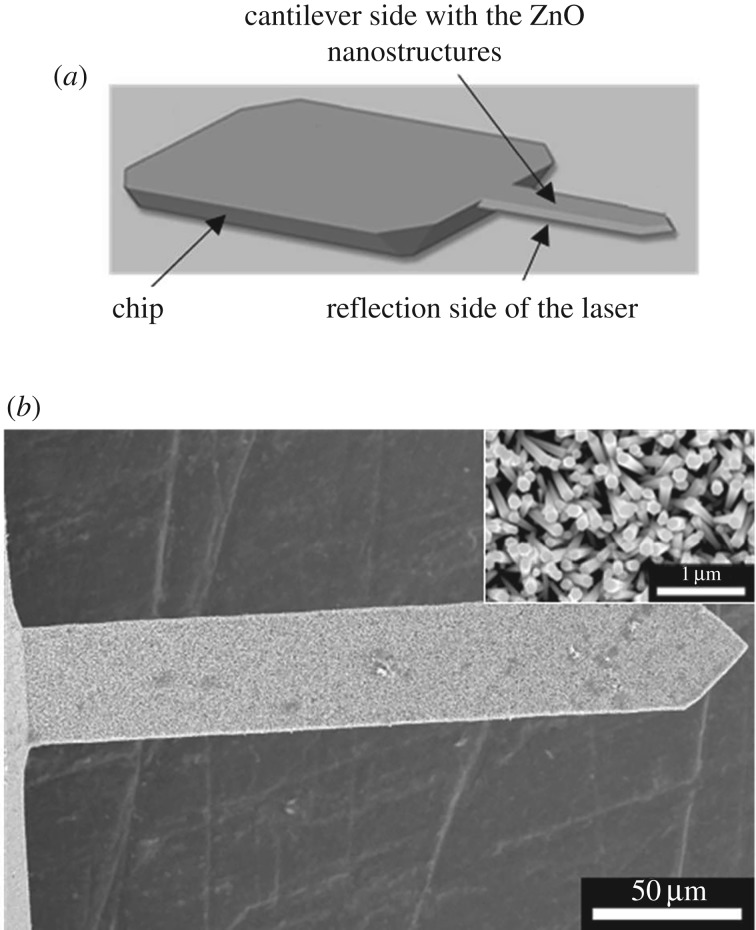
(*a*) Diagram of an AFM cantilever with optical readout. (*b*) SEM pictures of the reflection side of an AFM cantilever after the synthesis of ZnO nanorods on the surface of the ZnO thin layer which was previously sputtered on the other face of the cantilever. Inset: zoom of the cantilever reflection side after the ZnO nanorod growth.

The syntheses of ZnO nanorods/nanotubes developed on silicon wafers were done on AFM cantilevers. The first step of the synthesis consists in the sputtering of a thin zinc oxide layer on the cantilever surface. During the sputtering, the chip of the cantilever was fixed to the sample holder with the laser reflection side of the cantilever facing the sample holder. The other face of the cantilever was facing the zinc oxide target. It is important to note that the cantilever cannot be in contact with the sample holder (i.e. there is a small space (≈1 mm) between the reflection side and the sample holder) because of the geometry of the cantilever and of the chip (figure 8*a*). After the deposition of the thin zinc oxide layer, the ZnO nanorods were synthesized on the surface of this layer. Figure 8 shows the laser reflection side of the cantilever after the synthesis of ZnO nanorods.

No perfectly aligned zinc oxide nanorods are present on the laser reflection side of the cantilever. The presence of these nanostructures is a problem as this side has to be reflective in order to reflect the laser. During the deposition of the zinc oxide layer on the other face of the cantilever, a small quantity of zinc oxide condenses also on the reflection side because this side is not in contact with the sample holder.

In order to prevent the growth of zinc oxide nanorods on the reflection side of the cantilever, the zinc oxide seed layers present on this side of the cantilever were covered by a thin layer of gold before the growth of the nanorods (figure 1*c*). Theoretically, gold cannot be sputtered on the other side of the cantilever because this surface is at the same height as chip surface (figure 8*a*). So, when the chip is in contact with the sample holder, the cantilever surface is also in contact with it. Three gold thicknesses were tested: 1, 2 and 3 nm. After the gold deposition, the three samples were placed inside the autoclave in order to perform the hydrothermal synthesis allowing the growth of ZnO nanorods. The surfaces of the cantilevers after the ZnO nanorod growth are visible on electronic supplementary material, figure S2. A gold layer of 1 nm does not stop the growth of ZnO nanorods on the laser reflection side of the cantilever. The deposited thickness is not sufficient. For a gold thickness of 3 nm on the laser reflection side, the other side of the cantilever is not totally covered with ZnO nanorods (electronic supplementary material, figure S2). This is due to the presence of a thin layer of gold on the ZnO seed layer which prevents the nanorod growth. So, gold is all the same present on this side, despite the fact that it was protected by the sample holder. So, it was decided to sputter 2 nm of gold on the laser reflection side of the cantilever as it seems to be the best compromise.

**Figure 9.**
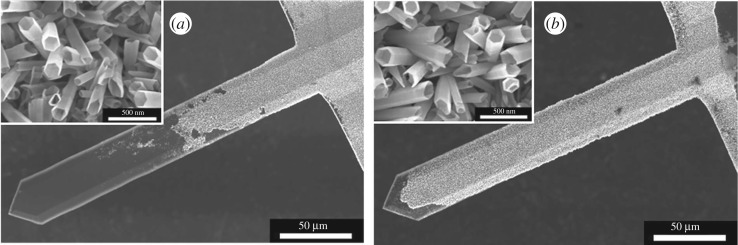
SEM top view of cantilever covered by ZnO nanotubes. These nanotubes are obtained by the dissolution during 1.75 h of ZnO nanorods in a solution containing (*a*) 0.75 wt% of CTAB and 0.75 wt% of NH_3(aq)_ and (*b*) 0.5 wt% of CTAB and 0.5wt% of NH_3(aq)_. Inset: zoom of the obtained nanotubes.

The nanorods present on the surface of the cantilevers were dissolved into nanotubes. On a wafer the best etching depth was obtained with a reaction of 1.75 h in an aqueous solution containing 0.5 wt% of NH_3(aq)_ and 0.5 wt% of CTAB. The volume of the solution was adapted to the cantilever surface, which has a surface 20 times lower than the used wafers (1 × 1 cm²). The volume of the solution is fixed to 4.5 ml. The obtained nanotubes are visible in figure 9*a*.

An important part of the cantilever surface is not covered with ZnO nanotubes. The nanotubes obtained in these etching conditions are well etched, but the seed layers are also drastically touched as only 50% of the cantilever is covered with nanorods. In order to cover all the cantilever, concentrations of the surfactant and of the etching agents were reduced. Both concentrations were fixed to 0.5 wt%. The etching result is visible in figure 9*b*. In these conditions, almost 95% of the cantilever surface is covered with ZnO nanotubes. The ZnO seed layer is also a little bit etched. Indeed, the dark part of figure 9*b* corresponds to a detachment of a part of the nanotubes. The etching depth of the nanotubes is higher (105.37 ± 5.68 nm; 10.4% of the total length) than the etching done in the same conditions on the wafer. This difference is due to the fact that the cantilever is on the border of the sample and that the diffusion of the dissolution reactants is better on the border of the samples. We tried also to increase the dissolution time for surfactants and etching agents’ concentrations equal to 0.5 wt%, but when the etching time increases, the cantilever surface covered with ZnO nanotubes decreases.

Kilinc *et al*. proved that ZnO nanostructured cantilevers can be used to detect selectively diethylamine and triethylamine [14]. The etching depth of the nanotubes optimized in this paper is really higher than that of the nanotubes presented by Kilinc *et al*. [14], so the sensitivity of the nanostructured sensors should be better. With the developed procedure, the nanostructures grow only on one side of the cantilever, which is not the case in Kilinc *et al.* works [37]. The growth on both sides of, not perfectly aligned, 1D nanostructures prevents a reflection of the laser and consequently a good measurement of the resonance frequency. This procedure can be used for all types of commercial cantilevers having an optical readout and by everybody who is not specialized in the production of home-made cantilevers.

## 4. Conclusion

Cantilevers with optical readout were nanostructured with zinc oxide nanotubes having an external diameter close to 100 nm and an average length close to 1 µm. The nanotubes synthesis was first optimized on the surface of silicon wafers before it was transferred to the cantilevers. After the synthesis of zinc oxide nanorods, the dissolution of the centre of the nanorods in order to obtain nanotubes was optimized. The concentrations of reactants and the dissolution time were modified in order to have the longest etching depth. After 1.25 h in a dissolution solution containing 0.75 wt% of NH_3(aq)_ and 0.75 wt% of CTAB, the longest etching depth was obtained (165 nm). Only a part of the 1D nanostructures were etched either because of the too small diameter of the nanostructures or because of a lack of defect in the (0001) plans.

After the transfer of the synthesis to the cantilevers, nanostructures were present on both sides of the cantilever, which prevents the reflection of the laser. In order to protect the reflection face of the cantilever, gold is settled on this face which prevents the nanostructure growth. After this protection step, the nanotubes are synthesized only on one face of the cantilever. The nanotubes cover all the surface of the cantilever and the etching depth is higher than that obtained when the nanotubes are synthesized on a wafer.

## Supplementary Material

Additional figures

## Data accessibility

All relevant data are within the paper and within the electronic supplementary material files.
