# Supplementary material for: Synthesis of zinc oxide nanorods or nanotubes on one side of a microcantilever
Source: R Soc Open Sci. 2018 Aug 8;5(8):180510. doi: 10.1098/rsos.180510 (PMC6124106; doi:10.1098/rsos.180510)
Supplement: Additional figures [file rsos180510supp1.docx]

**Supplementary information**

Figure S1: CTAB thermogravimetric curve.


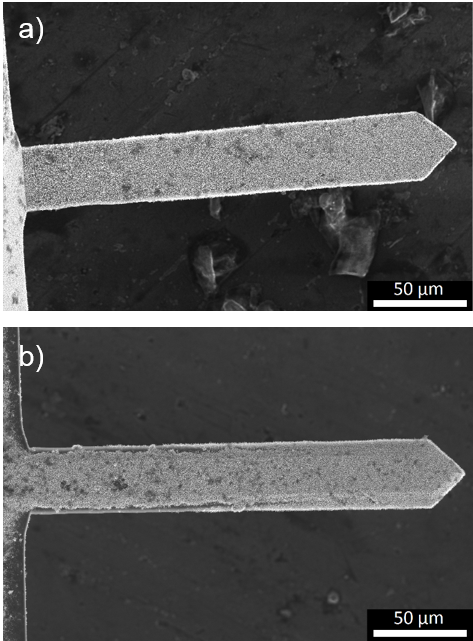


Figure S2: SEM top view a) of the LASER reflection side and b) of the other side, after the synthesis of ZnO nanorods. The LASER reflection side is covered with a) 1nm and b) 3 nm of gold before the nanorods growth.


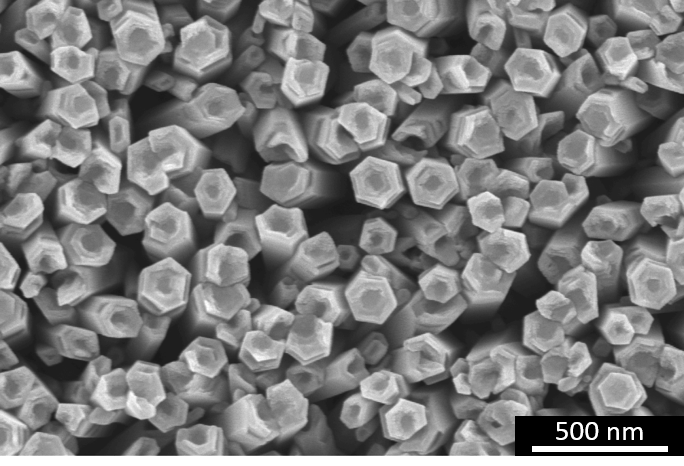


Figure S3: SEM top view of the nanotubes obtained after the dissolution during 3.5h of the nanorods in a solution containing 0.5 wt% of CTAB and 0.5 wt% of NH_3(aq)_. The nanorods were synthesized in an autoclave filled with 6 mL of ammonia (25 wt%) and with 80 mL of zinc chloride aqueous solution (0.1 mol/L). The autoclave is heated to 95°C during 70 min.
